# The influence of hospital leadership support on burnout, psychological safety, and safety climate for US infection preventionists during the coronavirus disease 2019 (COVID-19) pandemic

**DOI:** 10.1017/ice.2023.184

**Published:** 2024-03

**Authors:** Heather M. Gilmartin, Sanjay Saint, David Ratz, Kristin Chrouser, Karen E. Fowler, M. Todd Greene

**Affiliations:** 1 Denver/Seattle Center of Innovation for Veteran-Centered and Value Driven Care, Veterans Health Administration Eastern Colorado Healthcare System, Aurora, Colorado; 2 Department of Health Systems, Management and Policy, University of Colorado, Colorado School of Public Health, Aurora, Colorado; 3 VA Ann Arbor Healthcare System, Ann Arbor, Michigan; 4 Department of Internal Medicine, University of Michigan Medical School, Ann Arbor, Michigan; 5 Department of Urology, University of Michigan Medical School, Ann Arbor, Michigan; 6 University of Michigan/VA Ann Arbor Patient Safety Enhancement Program, Ann Arbor, Michigan

## Abstract

**Objective:**

To explore infection preventionists’ perceptions of hospital leadership support for infection prevention and control programs during the coronavirus disease 2019 (COVID-19) pandemic and relationships with individual perceptions of burnout, psychological safety, and safety climate.

**Design:**

Cross-sectional survey, administered April through December 2021.

**Setting:**

Random sample of non-federal acute-care hospitals in the United States.

**Participants:**

Lead infection preventionists.

**Results:**

We received responses from 415 of 881 infection preventionists, representing a response rate of 47%. Among respondents, 64% reported very good to excellent hospital leadership support for their infection prevention and control program. However, 49% reported feeling burned out from their work. Also, ∼30% responded positively for all 7 psychological safety questions and were deemed to have “high psychological safety,” and 76% responded positively to the 2 safety climate questions and were deemed to have a “high safety climate.” Our results indicate an association between strong hospital leadership support and lower burnout (IRR, 0.61; 95% CI, 0.50–0.74), higher perceptions of psychological safety (IRR, 3.20; 95% CI, 2.00–5.10), and a corresponding 1.2 increase in safety climate on an ascending Likert scale from 1 to 10 (β, 1.21; 95% CI, 0.93–1.49).

**Conclusions:**

Our national survey provides evidence that hospital leadership support may have helped infection preventionists avoid burnout and increase perceptions of psychological safety and safety climate during the COVID-19 pandemic. These findings aid in identifying factors that promote the well-being of infection preventionists and enhance the quality and safety of patient care.

The coronavirus disease 2019 (COVID-19) pandemic posed challenges for healthcare providers^1^ and hospital-based infection prevention departments.^2^ Infection preventionists, with backgrounds in nursing, public health, and microbiology, play a critical role in pandemic planning by communicating scientific guidelines. Initially, infection preventionists faced multiple challenges due to rapidly changing information, limited guidance for non–acute-care settings, and insufficient personal protective equipment (PPE) supplies.^3^ Over time, infection preventionists roles shifted from training staff to enforcing evolving policies like face shields and contact tracing, which were questioned for their effectiveness and purpose.^3^ This shift resulted in many infection preventionists reporting feeling a lack of control and a lack of credibility among staff.^3^

Infection prevention departments were positioned front and center during the pandemic and infection preventionists reported a 24-hour-a-day, 7-day-a-week workload that included their regular duties plus time-sensitive pandemic-related dilemmas. Such intensity undermined work–life balance.^4^ Additional challenges included the risk of exposure to the coronavirus at work and stigmatization of healthcare providers due to politicization of pandemic response plans and indecision from the Centers for Disease Control and Prevention (CDC). Many infection preventionists reported frustration with hospital leadership due to inadequate staffing on clinical units, financial restraints, or absent political support.^4^ These factors contributed to low infection-preventionist morale and reports of deteriorating mental and physical health and increasing reports of burnout.^3,5^

Infection preventionists are critical to patient safety due to their multifaceted roles as educators and implementers of infection prevention practices. Prior research suggests that effective efforts by infection preventionists requires leadership support, a psychologically safe environment, and a strong safety climate.^6–8^ Leadership support is expressed through the provision of adequate staffing, finances, and political support along with a commitment to a culture of excellence.^8^ Psychological safety is the shared belief that team members will not be reprimanded or punished for raising concerns or making mistakes.^6^ High psychological safety has been associated with improved team performance, well-being, and reduced burnout.^9^ Burnout is an occupational syndrome characterized by a high degree of emotional exhaustion, depersonalization, and a low sense of personal accomplishment at work.^10^ Safety climate is a subset of organizational climate that focuses on an individual’s perceptions about the extent to which the organization values safety.^7^ A safety climate in healthcare has been associated with better patient outcomes and enhanced employee well-being.^11^

Whether the response to the pandemic has affected how psychological safety and safety climate are perceived within the US healthcare system is uncertain, and the impact on infection prevention practices remains unknown. Numerous publications have highlighted the stress and burnout of healthcare workers^1,12,13^ and infection preventionists^2^ during the pandemic. However, few large-scale studies provide a picture of infection preventionist experiences during the pandemic that include data on organizational factors, such as hospital leadership support, psychological safety, safety climate, and infection preventionist well-being.^6–8^ In this study, we used data from a 2021 survey to determine (1) the state of hospital leadership support, infection preventionist burnout, psychological safety, and safety climate during the pandemic, and (2) relationships, mitigators, and aggravators among these variables.

## Methods

### Study design, survey instrument, and data collection

This national cross-sectional survey is part of an ongoing project in which, every 4 years since 2005, infection preventionists across the United States are asked about their hospital’s organizational characteristics and infection prevention practices.^14–20^ The 2021 population was based on a random sample of 900 general medical and surgical hospitals with an intensive care unit from the 2013 American Hospital Association database. Before finalizing the sample, an internet search was conducted to confirm each facility’s operating status. Closed facilities or those without general medical beds were removed from the mailing list. For the 2021 survey period, surveys were sent to a random sample of 883 nonfederal community hospitals. Prior to survey distribution, a letter was mailed addressed to the “infection control coordinator” at each nonfederal hospital notifying them of the forthcoming survey.

The initial survey was sent in mid-April 2021, and a reminder was mailed 2 weeks after initial survey distribution, requesting that the hospital infection preventionist (or supervising infection preventionist if there was more than one on staff) complete the survey. The survey could be completed on paper and returned by mail or electronically on a REDCap survey platform.^21^ Each survey was labeled with a unique study number that provided hospital identification; however, the individual respondents were anonymous. Additional reminder surveys were sent to nonresponding hospitals ∼1, 2, and 3 months following the initial survey distribution. Most surveys were returned by December 2021; just 3 were returned in early 2022. Two survey mailings were returned indicating that the hospitals had closed and were excluded from this analysis, leaving a total eligible sample of 881 nonfederal hospitals. Appropriate institutional review board exemption was obtained from the University of Michigan.

### Study measures

The survey assessed general hospital characteristics, including the number of acute-care beds, number of intensive care beds, affiliation with a medical school, patient care involvement of highest-ranking physician and nurse leaders in the organization, infection control program characteristics (ie, staffing, tenure, and certification in infection control and epidemiology), and leadership support for the infection control program. The survey also assessed infection preventionist perceptions of burnout,^22^ psychological safety,^23^ and safety climate.^24^ Strong leadership support was a derived dichotomous variable based on the 5-point Likert scale question, “How would you rank the overall support (eg, staffing, financial, and political) your infection prevention program receives from hospital administrative leadership?” We coded responses of 4 or 5 (very good or excellent) as 1, and 0 otherwise. A previously validated, single-item measure of emotional exhaustion, “I feel burned out from my work”^22^ and 7 psychological safety items were scored on a 5-point ascending Likert scale. Hospitals rating all 7 psychological safety questions as “agree or strongly agree” were deemed to have “high psychological safety” (coded as 1).^6^ Also, 2 questions within this domain were reverse scored so that values would represent positive responses.^6^ The 2 safety climate items were scored on a 5-point Likert scale. Scores were summed to create a safety climate score. Hand hygiene rates were queried using the single item, “What was the last overall hand hygiene compliance rate reported in your hospital (between 0 and 100%)?”

### Statistical analysis

Descriptive statistics were reported as percentages for categorical variables with means and standard deviations for continuous variables. For the burnout and psychological safety outcomes, dichotomous outcomes were modeled using Poisson regression with robust standard errors, incidence rate ratio (IRR) estimates, and 95% confidence intervals (CIs). The safety climate score outcome was modeled using linear regression, and β coefficients and 95% CIs. We conducted multivariable regressions to determine associations between hospital leadership support and perceptions of burnout, psychological safety, and safety climate. All models were adjusted for the following hospital characteristics: number of acute-care beds, medical school affiliation, hospital epidemiologist on staff, highest ranking physician and nurse direct patient care activity, and lead infection preventionist infection control certification status. If statistically significant in bivariable analysis with each outcome of interest, additional adjustments for the following respondent-specific characteristics were made: tenure in their position and tenure at the hospital. A *P* value <.05 was considered significant. All analyses were performed using SAS version 9.4 software (SAS Institute, Cary, NC).

## Results

The overall survey response rate was 47% (415 of 881). Select hospital and individual respondent characteristics are displayed in Table 1. There were no statistically significant differences in hospital characteristics between responding hospitals and nonresponders. The mean number of adult acute-care beds reported was 214. More than 34% of respondents reported working in hospitals affiliated with a medical school, 40% reported having a hospital epidemiologist on staff, and 69% indicated the lead infection preventionist was certified in infection control. The average number of full-time and part-time infection preventionists on staff was 2 and 0.3, respectively. In total, 88% of respondents reported having at least 1 full-time infection preventionist, 29% reported having at least 1 part-time infection preventionist, and 17% reported having at least 1 full-time and 1 part-time infection preventionist on staff. The average reported hand hygiene rate was 89%. More than 61% of respondents indicated that their highest-ranking physician (eg, chief medical officer, chief of staff) provided direct patient care, and only 9% reported direct patient-care activities for their highest-ranking nurse (eg, chief nursing officer or director of patient care services). Only 64% of respondents reported very good to excellent hospital leadership support for their infection prevention program. Most respondents reported their role as infection preventionist (85%), with an average of 8 years in their position and 14 years in their current hospital.

**Table 1.** Select Hospital and Individual Respondent Characteristics

Hospital Characteristic	Mean (SD)
Total no. of adult acute-care beds (including intensive care unit beds (n = 405)	214.2 (218.2)
Total number of adult intensive care unit beds (n = 408)	24.0 (32.6)
No. of full-time infection preventionists on staff (n = 402)	2.0 (2.2)
No. of part-time infection preventionists on staff (n = 402)	0.3 (0.7)
Hospital average hand hygiene rates (n = 390)	89% (11.7%)
	Frequency, No. (%)
Hospital is affiliated with a medical school.	141/412 (34.2)
Hospital has a hospital epidemiologist on staff.	161/399 (40.1)
Highest ranking physician (eg, chief medical officer, chief of staff) provides direct patient care.	251/414 (60.6)
Highest ranking nurse (eg, chief nursing office, director of patient care services) provides direct patient care.	36/414 (8.7)
Lead infection preventionist is certified in infection prevention and control.	276/402 (68.7)
Overall hospital leadership support (eg, staffing, financial, and political) for infection prevention program is very good or excellent.	266/413 (64.3)

Individual respondent characteristics	Frequency, No. (%)
Current position (n = 415)	
Infection preventionist	352 (84.8)
Nursing management	3 (0.7)
Hospital epidemiologist	11 (2.7)
Other	49 (11.8)
	Mean (SD)
Tenure in current position, y (n = 410)	8.3 (7.6)
Tenure in current hospital, y (n = 410)	14.0 (11.9)

Note. SD, standard deviation.

The responses to the 11 questions addressing burnout, psychological safety, and safety climate are shown in Table 2. In total, 49% of respondents reported feeling burned out from their work. Only 6% of respondents indicated that mistakes were held against employees, and only 18% indicated that staff were too busy to invest time in improvement. More than 90% of respondents indicated that they assert their views on important issues, even though their supervisor may disagree, 77% reported feeling supported to bring up problems and tough issues, and 77% indicated feeling comfortable speaking up when they see a physician not clean his or her hands. When a medical error occurs, >90% reported that their hospital encouraged employees to discuss mistakes to learn how to prevent similar future errors, and 70% reported feeling safe to try something new in their hospital. Approximately 30% of all respondents reported positively for all 7 questions; thus, we deemed them to have high psychological safety. On a scale of 1–10 points, the mean for the safety climate score was 8.33, suggesting that most respondents felt favorably about the safety climate in their respective hospitals.

**Table 2.** Burnout, Psychological Safety, and Safety Climate Items Rated Agree and Strongly Agree

Survey Domain	Survey Item	Frequency, n/N (%)
Burnout	I feel burned out from my work.	195/401 (48.6)
Psychological safety	I assert my views on important issues, even though my supervisor may disagree.	369/402 (91.8)
	I personally feel comfortable speaking up when I see a physician not clean his or her hands.	311/402 (77.4)
	When a medical error occurs at this hospital, employees are encouraged to discuss mistakes to learn how to prevent similar future errors.	364/402 (90.5)
	If you make a mistake at this hospital, it is often held against you.^a^	25/402 (6.2)
	Employees at this hospital can bring up problems and tough issues.	311/402 (77.4)
	It is safe to try something new at this hospital.	280/402 (69.7)
	At this hospital, people are too busy to invest time in improvement.^a^	71/402 (17.7)
	High psychological safety^b^	125/402 (31.0)
		Mean (SD)
Safety climate	I would feel safe being treated here as a patient (n=402).	4.21 (0.84)
	Leadership is driving us to be a safety centered institution (n=401).	4.12 (0.80)
	Safety climate score (n=401)^c^	8.33 (1.44)

a Questions were reverse scored; responses of 4 or 5 were positive.

b High psychological safety: defined as answering all 7 questions positively (ie, responses of 4 or 5 on a 5-point Likert-scale question).

c Safety climate score: defined as the sum of the two 5-point Likert scale questions (1, strongly disagree to 5, strongly agree): “I would feel safe being treated here as a patient,” and “Leadership is driving us to be a safety centered institution.” Possible scores ranged from 2 to 10.

### Multivariable models

Associations from the multivariable models between hospital and individual characteristics and the burnout, psychological safety, and safety climate outcomes are shown in Table 3. Strong hospital leadership support for the hospital’s infection prevention program was significantly associated with lower burnout (IRR, 0.61; 95% CI, 0.50–0.74), higher perceptions of psychological safety (IRR, 3.20; 95% CI, 2.00–5.10), and safety climate (β, 1.21; 95% CI, 0.93–1.49). Having a hospital epidemiologist on staff was significantly associated with high safety climate (β, 0.39; 95% CI, 0.09–0.69). The lead infection preventionist being certified in infection control was significantly associated with higher burnout (IRR, 1.33; 95% CI, 1.02–1.75). Also, infection preventionist tenure for every 5 years in their current position was significantly associated with higher burnout (IRR, 1.07; 95% CI, 1.01–1.13). Conversely, infection preventionist tenure for every 10 years working at the same hospital was significantly associated with a higher safety climate (β, 0.13; 95% CI, 0.02–0.24). Larger hospitals were significantly associated with higher burnout (IRR, 1.07; 95% CI, 1.02–1.12) and lower safety climate (β, −0.09; 95% CI, −0.16 to −0.02). There were no statistically significant associations with the outcomes of interest for medical school affiliation, or for the highest-ranking physician and nurse providing direct patient care.

**Table 3.** Adjusted Associations With Outcomes

Variables	IRR (95% CI)	*P* Value
Burnout (n = 380)^a^		
Strong hospital leadership support	0.61 (0.50–0.74)	**<.001**
Hospital epidemiologist	0.93 (0.74–1.16)	.53
Certified lead infection preventionist	1.33 (1.02–1.75)	**.04**
Time in position (per 5 years)	1.07 (1.01–1.13)	**.03**
No. of acute-care beds (per 100)	1.07 (1.02–1.12)	**.006**
Affiliated with a medical school	0.90 (0.71–1.13)	0.36
Highest ranking physician provides direct patient care	1.12 (0.91–1.37)	0.29
Highest ranking nurse provides direct patient care	1.35 (0.92–1.98)	0.12
Psychological safety (n = 386)^b^		
Strong hospital leadership support	3.20 (2.00–5.10)	**<.001**
Hospital epidemiologist	1.12 (0.82–1.54)	0.47
Certified lead infection preventionist	1.01 (0.72–1.41)	0.95
No. of acute-care beds (per 100)	0.96 (0.88–1.03)	0.26
Affiliated with a medical school	0.92 (0.66–1.29)	0.65
Highest ranking physician provides direct patient care	1.25 (0.91–1.73)	0.17
Highest ranking nurse provides direct patient care	1.04 (0.67–1.61)	0.86
Safety climate (n = 380)^c^	ß (95% CI)	
Strong hospital leadership support	1.21 (0.93 to 1.49)	**<.001**
Hospital epidemiologist	0.39 (0.09 to 0.69)	**.01**
Certified lead infection preventionist	0.01 (−0.31 to 0.33)	.96
Time at hospital (per 10 years)	0.13 (0.02 to 0.24)	**.02**
No. of acute care beds (per 100)	−0.09 (−0.16 to −0.02)	**.02**
Affiliated with a medical school	0.02 (−0.29 to 0.33)	.88
Highest ranking MD provides direct patient care	0.08 (−0.20 to 0.36)	.57
Highest ranking RN provides direct patient care	−0.37 (−0.87 to 0.13)	.15

Note. IRR, incidence rate ratio. β, beta coefficient. Bold indicates statistical significance.

a Burnout was defined as answering positively to the burnout question in Table 2.

b High psychological safety was defined as answering positively for the 7 questions presented in Table 2.

c High safety climate was defined as answering positively for the 2 questions presented in Table 2.

## Discussion

In our national, cross-sectional survey, we discovered that having strong hospital leadership support (defined in this study as adequate staffing, financial, and political support) for the hospital’s infection prevention program was significantly associated with lower rates of infection preventionist burnout and increased perceptions of psychological safety and safety climate. These findings are consistent with previous research by Zhou et al^25^ (which demonstrated the protective effects of organizational support on healthcare workers during epidemics using the Job Demands–Resources model) and a review by Schneider et al.^26^ (which identified organizational support as being protective of the well-being of healthcare workers responding to global pandemics). The next step in this research is to identify specific hospital leadership activities^27^ that individuals and organizations can implement to enhance the well-being of infection preventionists after the pandemic.

The 2022 US Surgeon General report^12^ outlined multiple leadership actions to build a thriving healthcare workforce. These actions include ensuring safe staffing levels, increasing access to mental health care for clinicians, reducing clinical administrative burdens, and promoting diversity among healthcare workers. Additional recommendations include establishing a chief wellness officer, incorporating well-being metrics into performance indicators for the organization, and linking executive compensation with improvements in healthcare worker well-being.^12^ Implementing such system-level changes will require leaders at all levels to model the desired behaviors and regularly communicate their commitment to these principles. Healthcare leaders can start by enhancing leadership and staff interactions through structured rounding, interactive huddles, and embracing difficult conversations.^27^ They can also lead with kindness, compassion, and love.^27^ Evidence-based practices from organizations like the Institute for Healthcare Improvement^28^ and the National Academy of Medicine^13^ can provide additional guidance for leaders seeking to create a culture that prioritizes the health, well-being, and safety of their workforce.

According to our national survey, 49% of respondents reported feeling burned out from their work, which is lower than the 65% reported by infection preventionists in a study by Melnyk et al^2^ during COVID-19 that used the following single burnout item: “Overall, based on your definition of burnout, how would you rate your level of burnout?” Even before the pandemic, the National Academy of Medicine found that burnout had reached crisis levels,^29^ with annual burnout-related turnover costs of $9 billion for nurses and $2.6–6.3 billion for physicians.^12^ Relevant to our findings, Linzer et al^12^ reported that the aggravators of burnout during COVID-19 included a lack of control over workload, chaotic workplace environments, challenges with teamwork, and a lack of feeling valued by organizations. Although individual-level solutions - such as yoga, meditation, reframing, and gratitude - are recommended to address burnout in healthcare workers,^30^ the findings of this study and those of Linzer et al^12^ underscore the need for both individual- and organizational-level solutions to prevent burnout. These solutions include hospital leaders who actively and respectively listen to frontline workers, make changes based on feedback, and provide adequate staffing, financial, and political support.

Psychological safety is essential for fostering a culture of open communication and constructive feedback, which can result in better patient care and outcomes.^9^ In this study, we found a positive relationship between strong hospital leadership support and higher perceptions of psychological safety. Previous research in infection prevention has also shown a relationship between psychological safety, leadership, and use of infection prevention practices.^6,31^ It is concerning that our study found that infection preventionists feel more comfortable expressing their opinions on important issues than reporting instances of physicians not cleaning their hands. Correcting poor hand hygiene practice is crucial feedback for the individual and can help establish social norms and can discourage erroneous or intentional violations of hand hygiene.^32^ Speaking up about hand hygiene is a critical part of the infection preventionist’s job, but reluctance to do so can be attributed to the risk of damaging social relationships and power hierarchies between physicians and infection preventionists.^32^ To cultivate psychological safety, it is crucial to have support from leadership. When leaders demonstrate strong support for infection prevention activities, such as speaking up about hand hygiene, it can have a positive impact on the well-being and perceptions of a safety climate among infection preventionists.

Perceptions of safety climate and psychological safety are interrelated and form a critical foundation for promoting patient safety and employee well-being. Traditionally, these topics have been examined independently.^33^ The rates of leadership support and perceptions of psychological safety and safety climate found in this study were similar to those reported by respondents in our 2017 survey.^6^ Additionally, we confirmed that strong leadership support was also statistically significantly associated with increased perceptions of psychological safety and safety climate in 2017. Since this finding was also noted in the 2021 results, further promotion of dedicated leadership efforts to communicate that safety is a top priority and ensuring psychological safety between infection prevention and clinical teams appears to be warranted. A recent review summarized the 4 areas in which leadership can improve psychological safety: inclusiveness (welcoming and valuing member contributions); trustworthiness (promoting an environment of respect wherein members exhibit trust towards leadership); change oriented (empowering members to influence change without fear of reprisal); and ethical (high moral standards, truthfulness).^34^ Concerted efforts by hospital leadership to focus and iteratively improve these areas may lead to improvements in safety climate and effective teamwork within infection prevention programs, subsequently leading to improvements in patient care and employee well-being. Burnout was not assessed in the 2017 survey.

This study had several limitations. Although the response rate for our national survey was reasonable considering the pandemic, it was lower than previous survey administrations. We relied on self-report from the lead infection preventionist, nurse manager, or hospital epidemiologist at each hospital. As such, our findings may not reflect the overall views or beliefs of hospital employees and may not be generalizable to other departments. Although we surveyed ∼10% of all US hospitals and employed a sampling strategy to obtain a nationally representative sample, the hospitals choosing to participate may differ from those choosing not to participate, and staff suffering from burnout may be less likely to respond to surveys, affecting generalizability. Because of the cross-sectional nature of the study, causality cannot be inferred; however, the strength of the associations detected supports the meaningfulness of leadership support in relation to our outcomes. Finally, the hospital leadership support item may not have captured all aspects of the concept because several distinct issues (eg, staffing, financial, and political support) were combined into a single question, limiting our understanding of the most influential concept. This is also true for the burnout and psychological safety items, which are multidimensional concepts and were assessed through abbreviated survey questions.

Limitations notwithstanding, our results suggest that improving hospital leadership support is a critical organization-level solution that could meaningfully address the current challenges of high burnout and low psychological safety and safety climate for infection preventionists. Further research is needed to prospectively test interventions designed to build leadership support for infection prevention departments. We trust that these findings will increase attention to the role leadership support plays in infection preventionist well-being and ultimately the quality and safety of patient care.
